# Creating cloud platforms for supporting FAIR data management in biomedical research projects.

**DOI:** 10.12688/f1000research.140624.1

**Published:** 2024-01-03

**Authors:** Marcel Jentsch, Valentin Schneider-Lunitz, Ulrike Taron, Martin Braun, Naveed Ishaque, Harald Wagener, Christian Conrad, Sven Twardziok

**Affiliations:** 1 Berlin Institute of Health at Charité – Universitätsmedizin Berlin, Center of Digital Health, Berlin, 10117, Germany

**Keywords:** Cloud, data management, data science, bioinformatics, platforms, FAIR

## Abstract

Biomedical research projects are becoming increasingly complex and require technological solutions that support all phases of the data lifecycle and application of the FAIR principles. At the Berlin Institute of Health (BIH), we have developed and established a flexible and cost-effective approach to building customized cloud platforms for supporting research projects. The approach is based on a microservice architecture and on the management of a portfolio of supported services. On this basis, we created and maintained cloud platforms for several international research projects. In this article, we present our approach and argue that building customized cloud platforms can offer multiple advantages over using multi-project platforms. Our approach is transferable to other research environments and can be easily adapted by other projects and other service providers.

## Introduction

Utilizing cloud platforms to link researchers, combine data, and manage data throughout the entire data life cycle is advantageous for biomedical research initiatives. It has become common practice to employ big data analytics to stratify and identify biomarkers in life science research ranging from biodiversity, development, and diseases such as cancer, mental health, or rare diseases. The information pertaining to biological samples from an individual donor has become incredibly complex and may comprise various scientific domains, types, and levels of biological organization. ^1^ Examples include data from neuroimaging, genomics, proteomics, or even wearables and electronic health records. The different modalities of data can represent different aspects and levels of a complex biological system. Harmonization and integration of these diverse types of data within large research projects is a major challenge. ^1^ Managing the flow of large datasets within a project and between different processing and analysis steps creates many technical and regulatory challenges when data is distributed across institutes, states, and countries.

**Figure 1.**
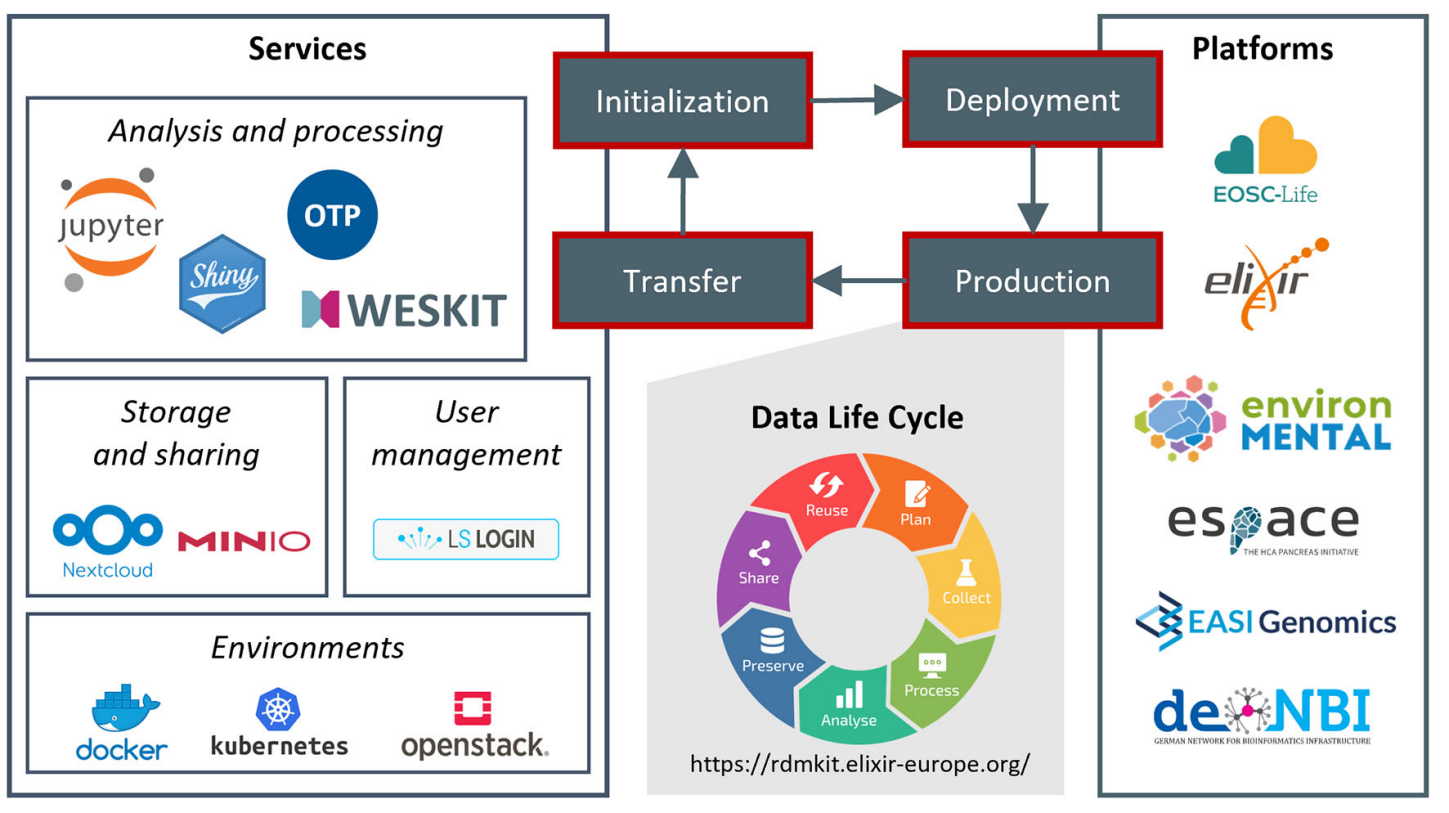
Platform life cycle. The schema describes the four phases of our platform development and project support. Starting with project initialization and selection of relevant services, we install a customized project platform in the deployment phase. In the production phase of the platform, projects use the services for data management activities covering the whole data life cycle. Finally, in the transfer phase, knowledge and experiences feed back into our service portfolio.

The data management requirements of research projects can be represented by the different phases of the data life cycle. The research community has access to numerous definitions of the data life cycle. Here, we adhere to the ELIXIR RDMkit’s ^2^ description, which classifies the data life cycle in research projects into the following seven phases ( Figure 1). Each phase has different requirements on technical solutions:

1. **Plan**: The planning phase involves the formalization of a data management plan. This very important and often mandatory document enables projects to organize data flows and processes within the project. Especially for larger projects, this phase requires the planning of data infrastructures that support data management during all the following phases of the data life cycle.
2. **Collect**: The collection phase involves performing multiple experiments and generation of data by e.g., application of instruments and running measurements. This phase requires technical solutions that connect the many different involved sites and move data to further processing facilities.
3. **Process**: Processing data sets typically requires the linking of several tools into a workflow that can transform input data into processed files. ^3^ An example is the alignment of Next Generation Sequencing (NGS) reads to a reference sequence followed by the detection of genomic variants. Popular workflow systems such as Nextflow ^4^ and Snakemake ^5^ support and structure the creation of workflows. Depending on their complexity, research projects may then require environments that manage and monitor many workflow executions.
4. **Analyze**: The analysis phase involves the use of applications, scripts, and notebooks to analyze data sets. Examples include applying machine learning methods, calculating statistics, summarizing data, or creating graphs. This task is usually performed interactively by an interdisciplinary, cross-institutional data science team. This requires an environment where different people can collaboratively access data as well as develop, share, and apply analysis scripts and notebooks.
5. **Preserve**: Data preservation is a critical process aimed at guaranteeing the long-term safety, integrity, and accessibility of data, spanning several decades if required. This covers a range of strategies and procedures to mitigate data loss risk, corruption, or obsolescence over time.
6. **Share**: Biomedical research projects often involve many partners from different institutes and countries. While accessing institutional computer centers is regularly restricted for external scientists, projects require solutions for sharing data between scientists. This also requires federated access functionalities.
7. **Re-use**: Providing computational access to data facilitates integration of the project data with external data sets from the external communities and reanalysis in new approaches.

Cloud technologies offer solutions for research data management by providing scalable resources for data processing, enabling controlled access to data through federated user management, and facilitating collaborative and interactive data analyses through shared workspaces. ^6^ There are different approaches to implement cloud platforms for biomedical research projects and a basic distinction is made between cloud types and service models. ^7^ Centralized platforms based on the System as a Service (SaaS) model provide data processing or data management functionalities for many users and multiple research projects in parallel. Hereby the platforms offer many established tools for the end-users, which support data management and can be used to answer project-specific questions. Important examples of multi-user platforms are Galaxy-Europe ^8^ and Anvil. ^9^ The creation of customized platforms that are subsequently run on a cloud Infrastructure as a Service (IaaS), or Platform as a Service (PaaS) architecture is a counter design to the centralized platforms.

Customized platforms that adhere to a modular microservice design can strengthen the FAIR principles and improve the interoperability of the research data by providing Application Programming Interfaces (APIs) for access to data slices and data summaries. ^10^ The FAIR principles are a set of guidelines for making data Findable, Accessible, Interoperable, and Reusable. ^11^ These principles aim to improve the ability of researchers and other stakeholders to locate, understand, and (re-) use data, which is of importance within large projects as well as for sharing data with the scientific community. Although it has many advantages, creating customized cloud platforms is a complex task and requires expert knowledge, experienced technical staff, and guidelines.

Here, we present our approach for creating customized cloud platforms for biomedical research projects and argue that such platforms offer many advantages for managing and processing data over the whole data life cycle and according to the FAIR principles. With similar use cases in mind, the framework is easily adaptable to various research initiatives and infrastructures. Our methodology offers a flexible framework for the management of knowledge and experiences while enabling the effective creation of cloud platforms.

### Approach

Our approach is based on managing a portfolio of supported services, which we have established within our cloud infrastructure. Our cloud is operated as a part of the German de. NBI network, ^12^ but this approach applies as well to other commercial or public providers. For our portfolio services, we document best practices, deployment scripts and default settings internally using our instance of GitLab. Using git enables collaborative file editing, allows versioning, and supports integration of deployment pipelines. We also provide general guidelines and best practices for the research community in the de. NBI Cloud Wiki ( https://cloud.denbi.de/wiki/). For developing our platforms, we follow a microservices architecture and manage our services as container images, which supports deploying these services in container environments. Our cloud environment mainly supports the container environments Docker and Kubernetes, which we apply depending on the requirements of the respective services.

During project initialization ( Figure 1), we consult projects, analyze which services are relevant for the respective project and design a platform framework. The consultation is generally performed in close coordination with the project management and scientists. The project-specific services are then installed on a cloud infrastructure in the deployment phase. We then maintain the project platforms throughout the whole production phase to support the data life cycle in the projects. Finally, knowledge and experiences of a project are transferred back into the portfolio and are then available for any other new project initialization. Hereby, we update deployment scripts and best practices as well as add new services, versions, and functionalities to our documentation. Applying this approach results in an evolving set of parallel running platforms over time, where any new platform profits from experiences and knowledge from all previous projects.

### Services

Our service portfolio consists of a set of core services ( Figure 1), which are regularly required by our collaboration partners. The selection of these services is based on our experiences and reflects the requirements of our research bubble. Anyhow, the general concepts can be easily transferred to other tools and services. Here, we describe how these services contribute to supporting the data life cycle and to the application of the FAIR principles. We generally recommend our projects to validate the application of FAIR criteria through self-assessment, as e.g., applied by de. NBI network. ^13^

*OpenStack Manila*

In most projects, we provide a shared network files system in our OpenStack cloud platform using the Manila service such as used in other OpenStack implementations. ^14^ All project-relevant data can be stored, managed, and shared in such a central file system. The other services can thus access the same database. This allows files to be shared with the scientists, who can access the data via the respective other services. The central data structure also ensures that the data can be clearly identified via paths within the project, which facilitates communication, increases the reproducibility of the results, and ensures the findability of the data.

*LS Login*

Life Science Login (LS Login) is a service which is developed by EOSC-life including the ELIXIR infrastructure to provide a unified user management and authorization infrastructure for European researchers. ^15^ It enables researchers to use their home organization credentials, community or other identities (e.g. Google, LinkedIn) to sign in and access data and services they are authorized to access. Thereby, it supports the OIDC standard which can easily be integrated by many other tools such as a user login system. By using LS Login users do not have to maintain multiple different user accounts for logging into the cloud platform services, which support the accessibility of data. By implementing LS Login, service providers don’t need to maintain their own user management system.

*JupyterHub*

JupyterHub is a multi-user platform, which allows for running Jupyter Notebook in a cloud environment. ^16^ Data scientists can use JupyterHub for interactive data analysis, and for sharing analysis scripts and results. Within the portal, the users have access to private home storage and optionally to shared cloud data, including the results of experiments, the shared user storage space, and a common code base. Optionally, JupyterHub can be configured to provide different compute resource profiles, such as high-memory machines for extensive data analysis and processing steps. Jupyter supports specifically the explorative analysis of research data.

*R-Shiny server*

R-Shiny is a framework that allows for rapid and intuitive development of interactive data visualization applications. We support serving of R-Shiny apps using the open-source Shiny Server. ^17^ R-Shiny apps can be used for interactive data analysis by members of a project as well as by external users. To ensure quality of services for multiple users, parallel processing over multiple servers as containers using Docker or Kubernetes is possible. A specific example of an R-Shiny app is the iSEE application, ^18^ which provides interactive analysis of single-cell data. The iSEE app can e.g., support annotation of cell types in single-cell data for members of a consortium by displaying gene expression in selected cell clusters. Providing interactive access to data supports data analysis and increases the accessibility of data for people without programming knowledge.

*MinIO*

Findability requires registration of metadata in public data registries and assigning unique identifiers to datasets. Using an S3 (Simple Storage Service) object storage, all files are structured into buckets and further grouped into folders. Single files or groups of files can then be identified via unique URIs. We usually set up an S3 endpoint via the open-source software MinIO. ^19^ The identifiers are globally unique since the domain name of the website is included. The unique identifier can then also be used to reference data in publications and in public data registries. An S3 server also provides an Application Programming Interface (API) access to the data, which allows access to all data that is to be published or shared with external users. The access to the S3 server can be public or protected by authentication.

*WESkit*

WESkit ^20^ is a workflow execution service implementing the GA4GH WES API. ^21^ It was developed to manage the execution of bioinformatics workflows at the German Cancer Research Center (DKFZ) and Charité Universitätsmedizin Berlin, but it can also be used in a cloud framework as well. The WESkit software provides features such as workflow monitoring, logging, and provenance tracking. It directly supports the processing of data. Hereby WESkit provides an interoperable environment for the execution of Snakemake ^5^ and Nextflow ^4^ workflows. The documentation of workflow executions supports the reusability of resulting data.

*OTP*

The platform “One Touch Pipeline” (OTP) was initially developed by the German Cancer Research Center (Deutsches Krebsforschungszenter, DKFZ) ^22^ as a part of the International Cancer Genome Consortium (ICGC) for management and processing of sensitive cancer genomics data. Since 2020, OTP is used at the DKFZ and Berlin Institute of Health for automatic execution of different workflows to process NGS data coming from whole genome sequencing (WGS), exome sequencing, RNA sequencing (RNA-seq), whole genome bisulfite sequencing (WGBS), and single-cell RNA sequencing (10x scRNA-seq) data. OTP has also been used for management of single-cell genomics data from COVID-19 patients. ^23^

*Nextcloud*

Nextcloud is a suite of client-server software for creating and using file hosting services. It is enterprise-ready with comprehensive support options. Being free and open-source software, anyone is allowed to install and operate it on their own private server devices. ^21^ It combines the comfort and user-friendliness of cloud solutions such as Dropbox or Google Drive with the requirements for security, data protection and control. The service runs on our infrastructure so that data does not need to leave the house. This allows users to share, for example, intermediate results and analysis scripts as part of data analysis. Access is granted to all members of a project and each user receives an initial 50 GB of storage.

### Implementation in projects

Multiple projects implemented the presented approach for creating customized cloud platforms to support the daily data life cycle.

*ESPACE*

The ESPACE project merged three prior Human Cell Atlas (HCA) early pilot studies to build a first version of the Human Cell Atlas of the Pancreas. The HCA project is an international effort to map all the cells in the human body and understand their functions. ^24^ The ESPACE cloud platform consists of shared storage, a JupyterHub portal, an R-Shiny Server for providing interactive applications and MinIO as an S3 backend for providing computational access to the data. The JupyterHub portal ( https://espace-cloud.bihealth.org) is actively used by more than ten data scientists throughout Europe for data processing and interactive data analysis. It is planned that the ESPACE data will soon be available to the research community via the ESPACE cloud platform.

*environMENTAL*

The environMENTAL project investigates how some of the greatest global environmental challenges, climate change, urbanization, and psychosocial stress affect mental health across the lifespan. The project aims to identify underlying molecular mechanisms and develop preventions and early interventions. Cohort data of over 1.5 million EU citizens and patients, enriched with deep phenotyping data from large-scale behavioral neuroimaging cohorts, are used to identify brain mechanisms related to environmental adversity underlying symptoms of depression, anxiety, stress, and substance abuse. The Berlin Institute of Health (BIH) ( https://www.bihealth.org/) provides a cloud platform to support data management in the environMENTAL project and all phases of the data life circle. The platform comprises a Nextcloud instance as a central storage for respective environmental data sets, a JupyterHub Portal as an interactive workspace and WESkit for running data processing on the integrated data sets.

*EASI-Genomics*

The EASI-Genomics consortium aims to provide translational access to cutting-edge sequencing technologies and data analysis methodologies to researchers, adhering to ethical and legal requirements, as well as FAIR and secure data management. A JupyterHub-based cloud platform was utilized to provide users with translational access to example datasets and Jupyter notebooks to provide guidelines for data analysis for single-cell, multi-omics and spatial transcriptomics data in both R and Python programming languages ( https://easi-genomics-cloud.bihealth.org).

*SpaceHack*

The JupyterHub-based cloud platform facilitated the SpaceHack project at the ELIXIR Germany BioHackathon 2022. The project’s objective was to generate benchmarking data sets by leveraging the expertise of researchers with both biological and technical backgrounds to evaluate the performance of segmentation and cell assignment tools in the context of tissue-specific challenges. ^25^ Over a week, the platform was utilized by more than 60 users, enabling virtual and onsite participants to collaborate effectively.

*OTP2EOSC*

The OTP2EOSC project developed a cloud-ready data management and processing platform that sends analysis workflows to the data, avoiding transferring data between sites. This increases the efficiency of data management and data security. The project deployed a demonstration cloud platform ( https://otp-demo.bihealth.org) for processing sensitive cancer-genomics data based on OTP, WESKit, and relevant ICGC cancer genomics workflows. The platform is available for interested users and to test the functionalities of the OTP software.

## Discussion

Collection, processing, analysis, storage, and access of biomedical research data require platforms for managing research data. ^26^ For this end, the use of a cloud platform can greatly support biomedical projects. There are already many existing platforms available and important examples of central multi-project platforms include Galaxy-Europe, ^8^ the AnVIL project, ^9^ the Human Cell Atlas data coordination platform ^24^ and the Cancer Genomics Cloud. ^27^ Such platforms can be used for specific use cases like the execution of Galaxy workflows or to work on specific data types like human cells or cancer genomics data. There are also several commercial providers including DNA Nexus and Seven Bridges, which offer the development and deployment of centralized multi-project platforms. Central platforms are generally operated via the SaaS model and the costs for infrastructure and resources are usually returned to the projects. ^7^ Alternatively, biomedical projects can also create their own customized platforms and run them using an IaaA or a PaaS approach.

From a data management perspective, there are several advantages and disadvantages to using a central multi-project cloud platform. These platforms come with a lot of functionality already built in and provide unified environments to support all stages of the data lifecycle, making it easier to define processes and train scientists across projects. However, biomedical scientists are typically involved in several different projects at the same time, and it can be challenging for them to work in many different cloud environments. ^10^ By using external central platforms, projects can worry less about technical infrastructure and have lower technical requirements. On the other hand, it can be a challenge to obtain funding for the use of external platforms ^7^ and integration into the local data access regulations and processes needs to be solved. Existing platforms do not necessarily provide all the desired functionality for specific projects and customization may be required. For multi-project platforms, there is a risk that changes and updates to individual components become more difficult when the number of ongoing projects increases, making it less flexible to respond to the needs of individual projects. Monolithic platforms also tend to suffer from lock-in effects and data becoming less interoperable. ^10^

The operation of a project-specific cloud platform based on an IaaS or PaaS model can offer several advantages over using a centralized multi-project framework. A customized cloud platform can save funding while operating on the existing resources of the institutes data centers, has greater flexibility and adaptability, and has a limited timeframe for long-term maintenance. Furthermore, long-term archiving may be easier to achieve. A local platform can be adapted to local data protection regulations such as those required by EU law with the General Data Protection Regulation (GDPR). On the technical side, copying large sets of data to a central platform can also be an issue. The features of a project-specific platform can be easily adapted to the specific needs of the users and the individual project goals. By developing self-controlled project-specific platforms, data interoperability and interconnection of platforms can be achieved more easily.

## Conclusions

A customized cloud platform can improve data sharing within a consortium, support data analysis and provide access to data over the whole data life cycle according to the FAIR criteria. This is particularly useful in an environment where many different partners within a consortium are analyzing a shared database. A common data structure also supports the sharing of analysis scripts and tools, making results reproducible within a project. In addition, data can be shared with external parties via a cloud platform as part of a review process, or more generally for reusing data by other researchers in new analyses. Hereby, our approach offers a structured and efficient way for developing platforms and serving multiple research projects.

The approach presented here of managing a portfolio of supported services has proven its worth in various projects at the BIH. A major benefit is the ease and speed with which new projects can be launched and supported. Due to management of know-how, existing platforms can be easily transferred to new projects within a few working days. By using existing software solutions and a microservice architecture, the individual components of the platform can be set up in a short time by a skilled cloud engineer. The microservices architecture makes the platform flexible and adaptable to individual user needs. In addition, the portfolio can be constantly updated during the transferring of services to new projects. However, this approach only makes sense if the potential projects have recurring software and service requirements. It is also a challenge to manage, update, extend and clean up the service portfolio on an ongoing basis.

Our supported projects benefited greatly from the provision of the de. NBI cloud for research in Germany and its integration into the European ELIXIR network. The de. NBI cloud is a federated cloud framework operated by the de. NBI network in Germany and is available for academic projects in Germany. ^12^ The de. NBI cloud is involved in the European Open Science Cloud (EOSC) project via the ELIXIR network with other European partners. Through EOSC, academic partners across Europe can also access cloud resources. The federated structure of the de. NBI cloud supports making platforms available where the data is stored. This has technical advantages with data transfer as well as legal advantages, e.g., if data is not allowed to leave an organization or to cross regional boundaries. It also has the advantage that local contact people are available on site. In addition, central services such as the central de. NBI project management system, established processes, and best practices as well as the LS Login can be used and accessed. The local availability of cloud technologies is of significant value for our project partners.

## Data Availability

No data are associated with this article.
