# Supplementary material for: Creating cloud platforms for supporting FAIR data management in biomedical research projects
Source: F1000Res. 2024 Apr 29;13:8. Originally published 2024 Jan 3. [Version 3] doi: 10.12688/f1000research.140624.3 (PMC11109697; doi:10.12688/f1000research.140624.3)
Supplement: Peer review history

# Reviewer report: Reviewer response for version 3

**DOI:** 10.5256/f1000research.165476.r270071

**Published:** 2024-05-09

**Authors:** Anna Bernasconi

**Affiliations:** 1 Politecnico di Milano, Milan, Lombardy, Italy

I confirm that my points have been addressed satisfactorily.

Is the topic of the opinion article discussed accurately in the context of the current literature?

Partly

Are arguments sufficiently supported by evidence from the published literature?

Yes

Are all factual statements correct and adequately supported by citations?

Yes

Are the conclusions drawn balanced and justified on the basis of the presented arguments?

Partly

Reviewer Expertise:

Data integration, Genomic data, Knowledge management, Bioinformatics, FAIR principles

I confirm that I have read this submission and believe that I have an appropriate level of expertise to confirm that it is of an acceptable scientific standard.

# Reviewer report: Reviewer response for version 2

**DOI:** 10.5256/f1000research.163890.r258433

**Published:** 2024-04-29

**Authors:** Joseph Bonello

**Affiliations:** 1 University of Malta, Msida, Malta

No further comments.

Is the topic of the opinion article discussed accurately in the context of the current literature?

Yes

Are arguments sufficiently supported by evidence from the published literature?

Yes

Are all factual statements correct and adequately supported by citations?

Yes

Are the conclusions drawn balanced and justified on the basis of the presented arguments?

Yes

Reviewer Expertise:

Bioinformatics; FAIR; Data Science

I confirm that I have read this submission and believe that I have an appropriate level of expertise to confirm that it is of an acceptable scientific standard.

# Reviewer report: Reviewer response for version 2

**DOI:** 10.5256/f1000research.163890.r258432

**Published:** 2024-04-10

**Authors:** Anna Bernasconi

**Affiliations:** 1 Politecnico di Milano, Milan, Lombardy, Italy

I appreciate the changes made to Fig 1, which I think now can better support readers throughout the explanation. I think that the "cleaning" of nomenclature will benefit the readability of the contribution. I appreciate the authors revising the Services' descriptions.

 I understand that, as this is an opinion article, the authors decided to focus on their own experience, however, I still believe that some discussion/take-home-messages section, with teachings on authors' own experience compared to others in the domain, would largely benefit the impact of this contribution. 

 Apart from this, I am convinced of the value of the article, which can be approved after this small addition.

Is the topic of the opinion article discussed accurately in the context of the current literature?

Partly

Are arguments sufficiently supported by evidence from the published literature?

Yes

Are all factual statements correct and adequately supported by citations?

Yes

Are the conclusions drawn balanced and justified on the basis of the presented arguments?

Partly

Reviewer Expertise:

Data integration, Genomic data, Knowledge management, Bioinformatics, FAIR principles

I confirm that I have read this submission and believe that I have an appropriate level of expertise to confirm that it is of an acceptable scientific standard, however I have significant reservations, as outlined above.

# Response

**Published:** 2024-04-23

**Authors:** Sven Twardziok

**Affiliations:** Berlin Institute of Health at Charité – Universitätsmedizin Berlin, Berlin, Berlin, Germany

We agree that the article lacked a final conclusion on how to apply our experience to external teams. In the new version, we have added a final conclusion and contrasted it with alternative approaches and platforms, which were already discussed in the Discussion section. We have also changed a passage in the text to emphasize the generalisability of the approach.

# Reviewer report: Reviewer response for version 1

**DOI:** 10.5256/f1000research.153998.r242776

**Published:** 2024-03-06

**Authors:** Joseph Bonello

**Affiliations:** 1 University of Malta, Msida, Malta

**Summary:**

 The project is about using a local microservice-based cloud architecture as a cost-effective solution over a per-project platform to support FAIR data management. The authors highlight the tools thay have available in their environment and who how projects can leverage this technology with the benefit of having project-specific expertise, built over several projects, available to them.

 These are my detailed comments for the individual sections of the article.

**Abstract:**

 The abstract provides a summary of the project and highlights what the article is about sufficiently well.

**Introduction:**

 The introduction describes the use of cloud platforms in biomedical research. In particular, reference is made to the need for the analysis of large datasets that need to adhere to strict legal and ethical frameworks. The authors adhere to the ELIXIR RDMkit definition of the data lifecycle which they summarise for the benefit of the overall narrative. The authors summarise well the problem associated with researchers' use of data and provide a good overview of the FAIR principles.

**Approach:**

 The authors describe the approach as being based on Docker and Kubernetes and how they provision services based on a core set of services that facilitate the research process based on the existing needs of their users.

 The authors then proceed to list and describe the list of services they make available on the platform they maintain. To complement this list, they provide several examples of projects where the technologies have been implemented.

*Comments*:

 1. The authors mention the validation of adherence to FAIR principles through a self-assessment. It would be interesting if this self-assessment is defined better, to highlight the synergy between the provided services and adherence to the FAIR principles.

 2. The list of tools and services provided on the platform is interesting. However, how these tools relate to the FAIR principles, and to which principles they relate, is not immediately clear. I suggest that the authors explicitly link the tool to the relevant FAIR principle for added clarity.

**Discussion:**

 In the discussion section, the authors compare the proposed solution to multi-project platform, comparing the pros and cons of the two different solutions.

***Comments:***

 1. In terms of costs, comparing the costs between the multi-project solution and using an in-house solution needs to consider not only the cost of purchasing and maintaining the hardware (including the salary costs, power, etc). It is important to acknowledge these costs, although I am aware that they may be difficult to quantify and compare.

 2. I think that a short mention of the performance differences offered by the two solutions merits a mention. This is in view of the fact that in order to achieve high performance locally, you must acquire the hardware to match the needs (e.g. GPUs). This can be expensive and require expertise to maintain.

**Conclusion:**

 In the concluding remarks, the authors highlight the benefits of having a local data structure that supports sharing resources within the project. They highlight the benefits of the proposed solution and provide examples where the solution has been used.

**General suggestions for improvement:**

 - Capitalize machine learning (Machine Learning) and data science (Data Science).

 - Provide a figure where each of the tools that support the data lifecycle is organized under the relevant heading of the RDMKit phases.

 - Provide more details on the security aspects that a local solution offers, how it compares to multi-project solutions and how it helps researchers align with legal requirements.

 - Show more clearly (with examples) how a local solution would encourage reproducibility of scientific study and how, in reality, can project groups share their data and research within a FAIR environment.

Is the topic of the opinion article discussed accurately in the context of the current literature?

Yes

Are arguments sufficiently supported by evidence from the published literature?

Yes

Are all factual statements correct and adequately supported by citations?

Yes

Are the conclusions drawn balanced and justified on the basis of the presented arguments?

Yes

Reviewer Expertise:

Bioinformatics; FAIR; Data Science

I confirm that I have read this submission and believe that I have an appropriate level of expertise to confirm that it is of an acceptable scientific standard.

# Response

**Published:** 2024-03-15

**Authors:** Sven Twardziok

**Affiliations:** Berlin Institute of Health at Charité – Universitätsmedizin Berlin, Berlin, Berlin, Germany

Dear reviewer,

 Thank you very much for your constructive comments and suggestions. Our reposes to your suggestions are in detail below and changes are included in the new version.

- The authors mention the validation of adherence to FAIR principles through a self-assessment. It would be interesting if this self-assessment is defined better, to highlight the synergy between the provided services and adherence to the FAIR principles.

We added an additional explanation of the term “self-assessment”. The reader will find more information in the cited publication.

- The list of tools and services provided on the platform is interesting. However, how these tools relate to the FAIR principles, and to which principles they relate, is not immediately clear. I suggest that the authors explicitly link the tool to the relevant FAIR principle for added clarity.

We modified the figure to make a connection between the phases of the data life cycle, the FAIR principles and the services.

- In terms of costs, comparing the costs between the multi-project solution and using an in-house solution needs to consider not only the cost of purchasing and maintaining the hardware (including the salary costs, power, etc). It is important to acknowledge these costs, although I am aware that they may be difficult to quantify and compare.

We added a sentence to make the reader aware of acquisition and operating costs; anyhow, our approach is not focused on running on institutes data centers, but an advantage is, that such existing hardware can be used.

- I think that a short mention of the performance differences offered by the two solutions merits a mention. This is in view of the fact that in order to achieve high performance locally, you must acquire the hardware to match the needs (e.g. GPUs). This can be expensive and require expertise to maintain.

The presented approach is not limited to the use of own hardware and therefore the purchase of own hardware is not a requirement.

# Reviewer report: Reviewer response for version 1

**DOI:** 10.5256/f1000research.153998.r242781

**Published:** 2024-02-22

**Authors:** Anna Bernasconi

**Affiliations:** 1 Politecnico di Milano, Milan, Lombardy, Italy

The paper describes the choice of adopting customized cloud platforms over centralized multi-project platforms in the context of biomedical research. The authors explain that, so far, the main trend has preferred the first kind of infrastructure instead of the second, bringing several limitations.

 I do believe that the author's experience is of great value and is worth being shared with a broader community. Therefore, I make some suggestions that should lead to improving the presentation of the work in the current manuscript.

 Figure 1 should drive the reader along the whole paper; however, it is not well-used. E.g. seven phases in the data life cycle are not connected to the other parts of the figure. The four project phases are not described in the paper. Services have been partitioned into four areas in the figures but this organization is not reflected in the paper (Services section, pages 5-6).

 Several parts of the Introduction and Discussion attempt to draw a line between centralized platforms based on SaaS (sometimes called central multi-project cloud platforms) and "customized cloud platforms" (based on IaaS/PaaS) such as the one proposed by the authors (see Page 4 and 7). This distinction is far from being well-explained and pragmatically justified. I urge the authors to better describe this, as it is fundamental for understanding their contribution. 

 The paper is focused on a specific experience, the one of the Berlin Institute of Health. The experience would be plausible in many other research institutes; however, the paper reads more like an experience paper/institute report rather than a research paper. This is even more evident when the author themselves speak about their research bubble ("The selection of these services is based on our experiences and reflects the requirements of our research bubble."). To overcome this, a more structured discussion on competitor / alternative platforms is needed. In this way, the paper would become understandable/useful also for whom is coming from different "bubbles".

 The descriptions of Services sometimes provide details that are not properly justified (e.g., why explain that 'Access is granted to all members of a project and each user receives an initial 50 GB of storage.' if further scalability is not discussed? Is 50GB a limitation in practical scenarios?)

 MINOR:

 - Page 5: explain OIDC acronym

 - don't --> do not

 - The adherence to FAIR principles is evaluated by "self-assessment". Could this be elaborated further?

 - Page 6, WESkit paragraph, remove 'as well'

 - Bibliography, please provide URLs for entries 2, 16, 17, 19, 25

Is the topic of the opinion article discussed accurately in the context of the current literature?

Partly

Are arguments sufficiently supported by evidence from the published literature?

Yes

Are all factual statements correct and adequately supported by citations?

Yes

Are the conclusions drawn balanced and justified on the basis of the presented arguments?

Partly

Reviewer Expertise:

Data integration, Genomic data, Knowledge management, Bioinformatics, FAIR principles

I confirm that I have read this submission and believe that I have an appropriate level of expertise to confirm that it is of an acceptable scientific standard, however I have significant reservations, as outlined above.

# Response

**Published:** 2024-03-15

**Authors:** Sven Twardziok

**Affiliations:** Berlin Institute of Health at Charité – Universitätsmedizin Berlin, Berlin, Berlin, Germany

Dear reviewer,

 Thank you very much for your constructive comments and suggestions. Our reposes to your suggestions are in detail below and changes are included in the new version.

- Figure 1 should drive the reader along the whole paper; however, it is not well-used. E.g. seven phases in the data life cycle are not connected to the other parts of the figure. The four project phases are not described in the paper. Services have been partitioned into four areas in the figures but this organization is not reflected in the paper (Services section, pages 5-6).

Thank you very much for this comment. We adapted the description of the four project phases the text. We also We modified the figure to make a connection between the phases of the data life cycle, the FAIR principles and the services.

- Several parts of the Introduction and Discussion attempt to draw a line between centralized platforms based on SaaS (sometimes called central multi-project cloud platforms) and "customized cloud platforms" (based on IaaS/PaaS) such as the one proposed by the authors (see Page 4 and 7). This distinction is far from being well-explained and pragmatically justified. I urge the authors to better describe this, as it is fundamental for understanding their contribution.

We agree that the distinction between the terms "centralised platforms" and "customised platforms" was unclear and have removed these distinctions accordingly throughout the manuscript. The manuscript now focuses more on the distinction between Iaas/PaaS and IasS at the project level, as these are common terms and projects ultimately have to choose between the models when building a cloud platform.

- The paper is focused on a specific experience, the one of the Berlin Institute of Health. The experience would be plausible in many other research institutes; however, the paper reads more like an experience paper/institute report rather than a research paper. This is even more evident when the author themselves speak about their research bubble ("The selection of these services is based on our experiences and reflects the requirements of our research bubble."). To overcome this, a more structured discussion on competitor / alternative platforms is needed. In this way, the paper would become understandable/useful also for whom is coming from different "bubbles".

We agree that this work reflects our own view. However, the framework of this work is an opinion paper and we believe that our experiences and the general concept in the present form will be useful for other readers as well.

- The descriptions of Services sometimes provide details that are not properly justified (e.g., why explain that 'Access is granted to all members of a project and each user receives an initial 50 GB of storage.' if further scalability is not discussed? Is 50GB a limitation in practical scenarios?)

​​​​​​Thanks for the hint. We have revised the description of the services and removed unnecessary information.

 MINOR:

- Page 5: explain OIDC acronym

We added an explanation of the OIDC acronym.

- don't --> do not

done

- The adherence to FAIR principles is evaluated by "self-assessment". Could this be elaborated further?

We added an additional explanation of the term “self-assessment”. The reader will find more information in the cited publication.

- Page 6, WESkit paragraph, remove 'as well'

done

- Bibliography, please provide URLs for entries 2, 16, 17, 19, 25

Urls are provided as links on the article website as well as in the pdf documents
